# (*E*)-1-(4-Chloro­phen­yl)-3-(4-methyl­phen­yl)prop-2-en-1-one

**DOI:** 10.1107/S160053680801324X

**Published:** 2008-05-10

**Authors:** Hoong-Kun Fun, Samuel Robinson Jebas, P. S. Patil, S. M. Dharmaprakash

**Affiliations:** a X-ray Crystallography Unit, School of Physics, Universiti Sains Malaysia, 11800 USM, Penang, Malaysia; b Department of Studies in Physics, Mangalore University, Mangalagangotri, Mangalore 574 199, India; c Department of Studies in Physics, Mangalore University, Mangalagangotri, Mangalore 574 199, India.

## Abstract

The title compound, C_16_H_13_ClO, adopts an *E* configuration with respect to the C=C double bond of the propenone unit. The dihedral angle between the two benzene rings is 45.9 (2)°. In the crystal structure, mol­ecules are arranged into sheets parallel to the *ac* plane and the sheets are stacked along the *b* axis. This arrangement is stabilized by weak inter­molecular C—H⋯π inter­actions involving both aromatic rings.

## Related literature

For applications of chalcones in non-linear optics, see: Agrinskaya *et al.* (1999 ▶). For related structures, see: Patil, Dharmaprakash *et al.* (2007 ▶); Patil, Fun *et al.* (2007 ▶); Patil, Rosli *et al.* (2007 ▶).
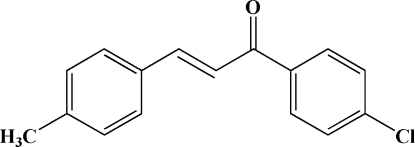


## Experimental

### 

#### Crystal data

C_16_H_13_ClO
*M* *_r_* = 256.71
Monoclinic, 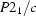

*a* = 15.2632 (3) Å
*b* = 14.0146 (3) Å
*c* = 5.8487 (1) Å
β = 92.154 (1)°
*V* = 1250.20 (4) Å^3^
*Z* = 4
Mo *K*α radiation
μ = 0.29 mm^−1^
*T* = 100.0 (1) K
0.34 × 0.18 × 0.05 mm

#### Data collection

Bruker SMART APEXII CCD area-detector diffractometer
Absorption correction: multi-scan (*SADABS*; Bruker, 2005 ▶) *T*_min_ = 0.908, *T*_max_ = 0.986
15494 measured reflections
3661 independent reflections
2534 reflections with *I* > 2σ(*I*)
*R*_int_ = 0.054

#### Refinement

*R*[*F* ^2^ > 2σ(*F* ^2^)] = 0.053
*wR*(*F* ^2^) = 0.129
*S* = 1.04
3661 reflections
164 parameters
H-atom parameters constrained
Δρ_max_ = 0.41 e Å^−3^
Δρ_min_ = −0.27 e Å^−3^

### 

Data collection: *APEX2* (Bruker, 2005 ▶); cell refinement: *APEX2*; data reduction: *SAINT* (Bruker, 2005 ▶); program(s) used to solve structure: *SHELXTL* (Sheldrick, 2008 ▶); program(s) used to refine structure: *SHELXTL*; molecular graphics: *SHELXTL*; software used to prepare material for publication: *SHELXTL* and *PLATON* (Spek, 2003 ▶).

## Supplementary Material

Crystal structure: contains datablocks global, I. DOI: 10.1107/S160053680801324X/ci2595sup1.cif

Structure factors: contains datablocks I. DOI: 10.1107/S160053680801324X/ci2595Isup2.hkl

Additional supplementary materials:  crystallographic information; 3D view; checkCIF report

## Figures and Tables

**Table 1:** Hydrogen-bond geometry (Å, °)

*D*—H⋯*A*	*D*—H	H⋯*A*	*D*⋯*A*	*D*—H⋯*A*
C9—H9⋯O1	0.93	2.50	2.820 (2)	100
C5—H5⋯*Cg*1^i^	0.93	2.98	3.525	119
C2—H2⋯*Cg*2^ii^	0.93	2.93	3.563	127
C14—H14⋯*Cg*2^iii^	0.93	2.80	3.495	132

Symmetry codes: (i) 
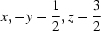
; (ii) 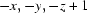; (iii) 
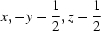
. *Cg*1 and *Cg*2 are the centroids of the C1–C6 and C10–C15 rings, respectively.

**Fig. 1.**
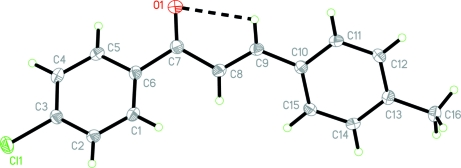
The molecular structure of the title compound, showing 50% probability displacement ellipsoids and the atom-numbering scheme. The intramolecular hydrogen bond is shown as a dashed line.

**Fig. 2.**
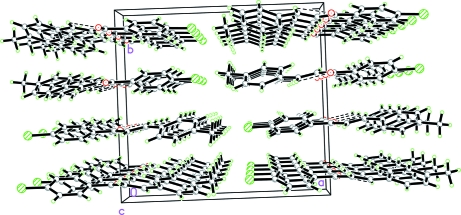
The crystal packing of the title compound, viewed along the c axis. Hydrogen bonds are shown as dashed lines.

## supplementary crystallographic information

### Comment

Recently, significant progress has been achieved in the growth of noncentrosymmetric crystals for second harmonic generation (SHG), mostly that of chalcone derivatives substituted with different donor/acceptor substituents (Agrinskaya *et al.*, 1999; Patil, Dharmaprakash *et al.*, 2007; Patil, Fun *et al.*, 2007; Patil, Rosli *et al.*, 2007). Herein we report the crystal structure of the title compound.

The title molecule (Fig.1) exhibits an E configuration with respect to the C8?C9 double bond, with the C7—C8—C9—C10 torsion angle being -175.3 (2)°. The bond lengths and angles are comparable to those observed in related structures (Patil, Dharmaprakash *et al.*, 2007; Patil, Fun *et al.*, 2007; Patil, Rosli *et al.*, 2007). The dihedral angle between the two benzene rings is 45.9 (1)°.

In the molecular structure, an intramolecular C—H···O hydrogen bond generates an S(5) ring motif. In the crystal structure, the molecules are arranged into sheets parallel to the *ac* plane and the sheets are stacked along the *b* axis (Fig. 2). This arrangement is stabilized by weak intermolecular C—H···π interactions involving both aromatic rings, Table 1.

### Experimental

The title compound was synthesized by the condensation of *p*-tolualdehyde (0.01 mol) with 4-chloroacetophenone (0.01 mol) in methanol (60 ml) in the presence of a catalytic amount of sodium hydroxide solution (5 ml, 30%). After stirring for 6 h, the contents of the flask were poured into ice-cold water (500 ml) and left to stand for 12 h. The resulting crude solid was filtered and dried. Single crystals suitable for X-ray diffraction were grown by slow evaporation of an acetone solution at room temperature.

### Refinement

H atoms were positioned geometrically and refined using a riding model with C—H = 0.93 Å for aromatic H and 0.96 Å for methyl H atoms. The *U*_iso_ values were constrained to be 1.5*U*_eq_ of the carrier atom for the methyl H atoms and 1.2*U*_eq_ for the remaining H atoms. A rotating-group model was used for the methyl group.

### Crystal data

**Table:**

C_16_H_13_ClO	*F*_000_ = 536
*M**_r_* = 256.71	*D*_x_ = 1.364 Mg m^−^^3^
Monoclinic, *P*2_1_/*c*	Mo *K*α radiation λ = 0.71073 Å
Hall symbol: -P 2ybc	Cell parameters from 2431 reflections
*a* = 15.2632 (3) Å	θ = 2.7–29.9º
*b* = 14.0146 (3) Å	µ = 0.29 mm^−^^1^
*c* = 5.8487 (1) Å	*T* = 100.0 (1) K
β = 92.154 (1)º	Plate, colourless
*V* = 1250.20 (4) Å^3^	0.34 × 0.18 × 0.05 mm
*Z* = 4	

### Data collection

**Table:**

Bruker SMART APEXII CCD area-detector diffractometer	3661 independent reflections
Radiation source: fine-focus sealed tube	2534 reflections with *I* > 2σ(*I*)
Monochromator: graphite	*R*_int_ = 0.055
*T* = 100.0(1) K	θ_max_ = 30.1º
φ and ω scans	θ_min_ = 1.3º
Absorption correction: multi-scan(SADABS; Bruker, 2005)	*h* = −21→21
*T*_min_ = 0.908, *T*_max_ = 0.986	*k* = −19→19
15494 measured reflections	*l* = −6→8

### Refinement

**Table:**

Refinement on *F*^2^	Secondary atom site location: difference Fourier map
Least-squares matrix: full	Hydrogen site location: inferred from neighbouring sites
*R*[*F*^2^ > 2σ(*F*^2^)] = 0.053	H-atom parameters constrained
*wR*(*F*^2^) = 0.129	*w* = 1/[σ^2^(*F*_o_^2^) + (0.0438*P*)^2^ + 0.9337*P*] where *P* = (*F*_o_^2^ + 2*F*_c_^2^)/3
*S* = 1.04	(Δ/σ)_max_ = 0.001
3661 reflections	Δρ_max_ = 0.41 e Å^−^^3^
164 parameters	Δρ_min_ = −0.27 e Å^−^^3^
Primary atom site location: structure-invariant direct methods	Extinction correction: none

### Special details

**Table:**

Experimental. The data was collected with the Oxford Cyrosystem Cobra low-temperature attachment.
Geometry. All e.s.d.'s (except the e.s.d. in the dihedral angle between two l.s. planes) are estimated using the full covariance matrix. The cell e.s.d.'s are taken into account individually in the estimation of e.s.d.'s in distances, angles and torsion angles; correlations between e.s.d.'s in cell parameters are only used when they are defined by crystal symmetry. An approximate (isotropic) treatment of cell e.s.d.'s is used for estimating e.s.d.'s involving l.s. planes.
Refinement. Refinement of *F*^2^ against ALL reflections. The weighted *R*-factor *wR* and goodness of fit *S* are based on *F*^2^, conventional *R*-factors *R* are based on *F*, with *F* set to zero for negative *F*^2^. The threshold expression of *F*^2^ > σ(*F*^2^) is used only for calculating *R*-factors(gt) *etc*. and is not relevant to the choice of reflections for refinement. *R*-factors based on *F*^2^ are statistically about twice as large as those based on *F*, and *R*- factors based on ALL data will be even larger.

### Fractional atomic coordinates and isotropic or equivalent isotropic displacement parameters (Å^2^)

**Table:**

	*x*	*y*	*z*	*U*_iso_*/*U*_eq_	
Cl1	−0.37993 (3)	0.37197 (4)	0.72301 (10)	0.02546 (15)	
O1	0.01956 (10)	0.38013 (12)	0.3019 (2)	0.0258 (4)	
C1	−0.12084 (13)	0.40835 (14)	0.7779 (3)	0.0177 (4)	
H1	−0.0797	0.4295	0.8878	0.021*	
C2	−0.20925 (13)	0.40915 (14)	0.8251 (3)	0.0178 (4)	
H2	−0.2279	0.4322	0.9643	0.021*	
C3	−0.26921 (13)	0.37513 (14)	0.6615 (3)	0.0178 (4)	
C4	−0.24374 (13)	0.34215 (14)	0.4505 (4)	0.0192 (4)	
H4	−0.2850	0.3196	0.3425	0.023*	
C5	−0.15563 (13)	0.34352 (14)	0.4044 (3)	0.0178 (4)	
H5	−0.1375	0.3225	0.2630	0.021*	
C6	−0.09327 (12)	0.37615 (14)	0.5676 (3)	0.0166 (4)	
C7	0.00093 (13)	0.37789 (14)	0.5041 (4)	0.0183 (4)	
C8	0.06927 (13)	0.37523 (15)	0.6898 (3)	0.0190 (4)	
H8	0.0541	0.3596	0.8377	0.023*	
C9	0.15291 (13)	0.39512 (14)	0.6470 (3)	0.0170 (4)	
H9	0.1641	0.4150	0.4992	0.020*	
C10	0.22861 (13)	0.38904 (13)	0.8071 (3)	0.0160 (4)	
C11	0.31013 (13)	0.42159 (14)	0.7374 (3)	0.0172 (4)	
H11	0.3144	0.4494	0.5939	0.021*	
C12	0.38450 (13)	0.41295 (14)	0.8791 (4)	0.0187 (4)	
H12	0.4378	0.4358	0.8299	0.022*	
C13	0.38079 (13)	0.37070 (14)	1.0938 (3)	0.0180 (4)	
C14	0.29931 (13)	0.33856 (14)	1.1634 (3)	0.0174 (4)	
H14	0.2953	0.3101	1.3063	0.021*	
C15	0.22434 (13)	0.34813 (14)	1.0242 (3)	0.0167 (4)	
H15	0.1708	0.3271	1.0757	0.020*	
C16	0.46114 (13)	0.35970 (16)	1.2478 (4)	0.0241 (5)	
H16A	0.5117	0.3523	1.1567	0.036*	
H16B	0.4549	0.3044	1.3428	0.036*	
H16C	0.4682	0.4153	1.3425	0.036*	

### Atomic displacement parameters (Å^2^)

**Table:**

	*U*^11^	*U*^22^	*U*^33^	*U*^12^	*U*^13^	*U*^23^
Cl1	0.0154 (2)	0.0311 (3)	0.0299 (3)	0.0011 (2)	0.00166 (19)	−0.0017 (2)
O1	0.0214 (7)	0.0393 (9)	0.0169 (8)	−0.0012 (7)	0.0016 (6)	0.0010 (7)
C1	0.0183 (9)	0.0173 (9)	0.0171 (10)	−0.0015 (8)	−0.0029 (8)	0.0006 (8)
C2	0.0194 (9)	0.0177 (10)	0.0162 (10)	0.0015 (8)	0.0004 (8)	−0.0004 (8)
C3	0.0154 (9)	0.0166 (9)	0.0214 (10)	0.0007 (8)	0.0010 (8)	0.0031 (8)
C4	0.0195 (10)	0.0183 (10)	0.0195 (10)	0.0000 (8)	−0.0047 (8)	−0.0006 (8)
C5	0.0214 (10)	0.0171 (9)	0.0148 (10)	0.0022 (8)	−0.0013 (8)	−0.0002 (8)
C6	0.0161 (9)	0.0150 (9)	0.0185 (10)	0.0014 (8)	−0.0011 (7)	0.0032 (8)
C7	0.0179 (9)	0.0178 (9)	0.0192 (10)	0.0005 (8)	0.0009 (8)	−0.0001 (8)
C8	0.0196 (10)	0.0214 (10)	0.0161 (10)	0.0019 (8)	0.0004 (8)	0.0023 (8)
C9	0.0194 (9)	0.0165 (10)	0.0152 (10)	0.0023 (8)	0.0017 (8)	0.0010 (8)
C10	0.0162 (9)	0.0147 (9)	0.0171 (10)	0.0035 (7)	0.0013 (7)	−0.0015 (8)
C11	0.0205 (10)	0.0168 (10)	0.0144 (10)	0.0018 (8)	0.0022 (8)	0.0012 (8)
C12	0.0153 (9)	0.0197 (10)	0.0212 (11)	−0.0007 (8)	0.0037 (8)	0.0010 (8)
C13	0.0171 (9)	0.0177 (9)	0.0191 (10)	0.0012 (8)	−0.0012 (8)	−0.0021 (8)
C14	0.0207 (10)	0.0185 (10)	0.0129 (9)	−0.0004 (8)	0.0010 (8)	0.0006 (8)
C15	0.0160 (9)	0.0167 (10)	0.0175 (10)	−0.0010 (8)	0.0033 (8)	−0.0018 (8)
C16	0.0175 (10)	0.0309 (12)	0.0235 (11)	0.0004 (9)	−0.0030 (8)	0.0027 (9)

### Geometric parameters (Å, °)

**Table:**

Cl1—C3	1.742 (2)	C9—C10	1.462 (3)
O1—C7	1.227 (2)	C9—H9	0.93
C1—C2	1.388 (3)	C10—C15	1.397 (3)
C1—C6	1.390 (3)	C10—C11	1.400 (3)
C1—H1	0.93	C11—C12	1.385 (3)
C2—C3	1.384 (3)	C11—H11	0.93
C2—H2	0.93	C12—C13	1.391 (3)
C3—C4	1.387 (3)	C12—H12	0.93
C4—C5	1.382 (3)	C13—C14	1.397 (3)
C4—H4	0.93	C13—C16	1.502 (3)
C5—C6	1.399 (3)	C14—C15	1.386 (3)
C5—H5	0.93	C14—H14	0.93
C6—C7	1.499 (3)	C15—H15	0.93
C7—C8	1.478 (3)	C16—H16A	0.96
C8—C9	1.340 (3)	C16—H16B	0.96
C8—H8	0.93	C16—H16C	0.96
			
C2—C1—C6	120.50 (19)	C10—C9—H9	116.4
C2—C1—H1	119.7	C15—C10—C11	118.07 (18)
C6—C1—H1	119.7	C15—C10—C9	122.91 (18)
C3—C2—C1	118.86 (19)	C11—C10—C9	118.95 (18)
C3—C2—H2	120.6	C12—C11—C10	120.97 (18)
C1—C2—H2	120.6	C12—C11—H11	119.5
C2—C3—C4	122.00 (18)	C10—C11—H11	119.5
C2—C3—Cl1	119.19 (16)	C11—C12—C13	121.12 (19)
C4—C3—Cl1	118.81 (15)	C11—C12—H12	119.4
C5—C4—C3	118.44 (19)	C13—C12—H12	119.4
C5—C4—H4	120.8	C12—C13—C14	117.82 (18)
C3—C4—H4	120.8	C12—C13—C16	121.66 (18)
C4—C5—C6	120.91 (19)	C14—C13—C16	120.52 (18)
C4—C5—H5	119.5	C15—C14—C13	121.47 (19)
C6—C5—H5	119.5	C15—C14—H14	119.3
C1—C6—C5	119.27 (18)	C13—C14—H14	119.3
C1—C6—C7	122.63 (18)	C14—C15—C10	120.54 (18)
C5—C6—C7	118.07 (18)	C14—C15—H15	119.7
O1—C7—C8	121.77 (18)	C10—C15—H15	119.7
O1—C7—C6	119.90 (18)	C13—C16—H16A	109.5
C8—C7—C6	118.32 (18)	C13—C16—H16B	109.5
C9—C8—C7	120.57 (19)	H16A—C16—H16B	109.5
C9—C8—H8	119.7	C13—C16—H16C	109.5
C7—C8—H8	119.7	H16A—C16—H16C	109.5
C8—C9—C10	127.16 (19)	H16B—C16—H16C	109.5
C8—C9—H9	116.4		
			
C6—C1—C2—C3	1.6 (3)	C6—C7—C8—C9	−166.97 (19)
C1—C2—C3—C4	−1.3 (3)	C7—C8—C9—C10	−175.30 (19)
C1—C2—C3—Cl1	177.78 (15)	C8—C9—C10—C15	9.1 (3)
C2—C3—C4—C5	0.1 (3)	C8—C9—C10—C11	−173.9 (2)
Cl1—C3—C4—C5	−179.00 (15)	C15—C10—C11—C12	0.4 (3)
C3—C4—C5—C6	0.9 (3)	C9—C10—C11—C12	−176.80 (18)
C2—C1—C6—C5	−0.7 (3)	C10—C11—C12—C13	0.8 (3)
C2—C1—C6—C7	177.19 (18)	C11—C12—C13—C14	−1.0 (3)
C4—C5—C6—C1	−0.6 (3)	C11—C12—C13—C16	179.17 (19)
C4—C5—C6—C7	−178.54 (18)	C12—C13—C14—C15	0.0 (3)
C1—C6—C7—O1	−155.5 (2)	C16—C13—C14—C15	179.90 (19)
C5—C6—C7—O1	22.4 (3)	C13—C14—C15—C10	1.1 (3)
C1—C6—C7—C8	25.5 (3)	C11—C10—C15—C14	−1.3 (3)
C5—C6—C7—C8	−156.65 (18)	C9—C10—C15—C14	175.76 (18)
O1—C7—C8—C9	14.0 (3)		

### Hydrogen-bond geometry (Å, °)

**Table:**

*D*—H···*A*	*D*—H	H···*A*	*D*···*A*	*D*—H···*A*
C9—H9···O1	0.93	2.50	2.820 (2)	100
C5—H5···Cg1^i^	0.93	2.98	3.525	119
C2—H2···Cg2^ii^	0.93	2.93	3.563	127
C14—H14···Cg2^iii^	0.93	2.80	3.495	132

Symmetry codes: (i) *x*, −*y*−1/2, *z*−3/2; (ii) −*x*, −*y*, −*z*+1; (iii) *x*, −*y*−1/2, *z*−1/2.
